# Glycosylation disorders in pediatric epilepsy: pathophysiology, imaging and precision therapy

**DOI:** 10.3389/fneur.2026.1878916

**Published:** 2026-08-19

**Authors:** Lijuan Fan, Yajun Shen, Jianjun Wang, Jing Gan

**Affiliations:** 1 Department of Pediatrics, West China Second University Hospital, Sichuan University, Chengdu, China; 2 Key Laboratory of Birth Defects and Related Diseases of Women and Children, Ministry of Education, Sichuan University, Chengdu, China; 3 WCSUH-Tianfu·Sichuan Provincial Children’s Hospital, Meishan, Sichuan, China

**Keywords:** epilepsy, excitation-inhibition balance, glycosylation disorders, neural network remodeling, precision therapy

## Abstract

Epilepsy is a common neurological disorder in children and about 30% of children with epilepsy develop drug-resistant epilepsy. Epilepsy resulting from genetic variations and structural brain abnormalities has been extensively studied. Based on the current evidence, however, glycosylation, a major post-translational modification on over 70% of human brain proteins, is necessary for the stability of neural networks. This article delves into the many aspects of glycosylation abnormalities in epilepsy, including impairing early neural development, affecting the transport and function of ion channels, disrupting synaptic receptor and vesicular transport, and exacerbating neuroinflammatory damage. At the same time, the relationship between glycosylation and epilepsy is bidirectional. Glycosylation disorders induce seizures and chronic seizures induce a glycomic remodeling of the brain. This forms a vicious cycle and becomes a “background mechanism” of susceptibility and drug resistance for epilepsy. Specific and individualized approaches to clinical management must be adopted, including substrate supplementation, the ketogenic diet, and new specific drugs, such as enzyme inhibitors, pharmacological chaperones, and gene therapy. Future studies should focus on combining multi-omics data with high resolution neuroimaging to gain insights into the spatiotemporal dynamics of glycosylation abnormalities; the development of more targeted drugs according to the level of post-translational modification; and the formulation of more precise intervention strategies involving pediatric patients.

## 1 Introduction

Epilepsy is a common neurological disorder in children, characterized by the occurrence of recurring and unpredictable seizures, which seriously affect learning, behavior and the quality of life. Even with the constant emergence of new antiepileptic drugs, about 30% of children ultimately become drug-resistant (1). Current studies of epilepsy etiology have mainly focused on structural defects, neurotransmitter imbalance and ion channelopathies. But, at present, there is evidence that post-translational modifications (PTMs) are essential for the stability of neural networks and regulation of neuronal activity. Molecularly, PTMs control a wide range of protein properties such as maturation, stability, intracellular localization and enzymatic activity (2, 3). Of these changes, glycosylation, particularly biosynthesis of N-linked, O-linked and glycosylphosphatidylinositol (GPI) anchor proteins, is a key mechanism impacting over 70% of human brain proteins (4).

Glycosylation is the enzyme-catalyzed, stepwise attachment of carbohydrate chains (glycans) to proteins and lipids. The most common and important forms are N-linked glycosylation and O-linked glycosylation. N-linked glycosylation is initiated in the endoplasmic reticulum by transfer of a dolichol-linked oligosaccharide to asparagine residues and remodeled in the Golgi. O-linked glycosylation connects sugar chains to protein serine or threonine residues through O-glycosidic bonds and mainly comprises mucin-type O-GalNAc glycosylation, O-mannosylation and O-GlcNAcylation. In addition, there is the biosynthesis of gangliosides and the assembly of GPI anchors. Defects in any of these pathways give rise to congenital disorders of glycosylation (CDG) (5).

CDGs are usually multisystem diseases and almost all types of CDG have been reported to have epilepsy. A 2022 overview of metabolic epilepsies estimated that glycosylation disorders represent about 13.5% of the roughly 600 metabolic diseases that can present with seizures (6). The incidence of epilepsy varies significantly among different subtypes. In PMM2-CDG, the most common N-glycosylation defect, the reported frequency of seizures ranges from 11% to nearly half. The differences stem from inclusion criteria, follow-up duration, and whether stroke-like episodes were included (7). ALG6-CDG is the second most common CDG subtype, of which 70% are associated with epilepsy, with approximately 22% of patients developing drug-resistance (8). In certain CDG subtypes, epilepsy is present in nearly all patients, such as ALG13-, ALG11-, SLC35A2-, and PIGA-CDG (9). In addition to epilepsy, abnormal muscle tone, muscle weakness, ataxia, developmental delay, behavioral abnormalities, facial dysmorphism, and multiorgan dysfunction are common complications of CDG. These findings underscore the importance of considering glycosylation in the diagnostic evaluation of early-onset, unexplained epilepsy, particularly when accompanied by multisystem involvement.

The significance of glycosylation in neurodevelopment and its neurodegenerative effects has been established, but systematic reviews on its important role in seizure generation and maintenance are still lacking (10–12). Glycosylation studies of pediatric epilepsy will help to understand the basic underlying disease mechanisms and may help to identify new biomarkers and precision therapeutic targets. The molecular basis of this is reviewed and the clinical significance of this for diagnosis and treatment is discussed.

## 2 Current research status of the association between glycosylation and epilepsy

### 2.1 Roles of glycoproteins in neural development

In the rapidly developing brains of children, the glycosylation process is highly dynamic. Specifically, glycoproteins and glycolipids on neural stem cell surface are essential for orchestrating differentiation, migration, and synaptic cleft signaling (2, 13). By modulating the activity of cell adhesion molecules (such as NCAM, L1CAM, and Contactin) and extracellular matrix components, glycosylation provides the precise cues for axon guidance and neuronal localization (13–15). In addition, the membrane localization and functional stability of synapse-associated proteins such as PSD-95 and neuroligin are strictly regulated by their glycosylation status, which directly influences the plasticity of synaptic transmission (15, 16). When neuronal migration and synaptic connections are impaired, the resulting instability in neural networks provides the structural basis for the onset of epilepsy.

Multiple key genes for N-glycosylation have been confirmed to be directly related to brain development. For example, mutations in the *DPAGT1* gene cause defects in the initial step of N-glycosylation, leading to extensive abnormalities in neural development, synaptogenesis, and cell adhesion by disrupting the Wnt signaling pathway (17). Mutations in the UDP-galactose transporter encoded by the *SLC35A2* gene result in defective galactosylation of glycoproteins, causing abnormal accumulation of related proteins in cell adhesion and axon guidance pathways, and further inducing neuronal heterotopia and cortical lamination disorders, clinically presenting as mild malformation of cortical development with oligodendroglial hyperplasia in epilepsy (MOGHE) (18). O-mannosylation on *α*-dystroglycan (α-DG) is initiated by the POMT1/2 complex and extended to form matriglycan; defects in this structure directly lead to cortical malformations (19).

As a dynamic and reversible modification, O-GlcNAcylation is regulated by the opposing actions of O-GlcNAc transferase (OGT) and O-GlcNAcase (OGA). This process maintains neural homeostasis by precisely balancing the proliferation and differentiation of progenitor cells, affecting nearly 50% of proteins involved in central nervous system development. For example, O-GlcNAc modification of MeCP2 contributes to dendritic growth, while modification of Notch1 is critical for the self-renewal of neural stem cells. Thus, mutations in OGT or OGA that impair the function of the enzymes are associated with severe neurodevelopmental defects (20). Mucin-type O-GalNAc glycosylation is also deeply involved in neurodevelopment; During the early stages of neural development in mouse embryos, ppGalNAc-T13 expression is significantly upregulated, and it initiates neural differentiation via mucin-type O-glycoprotein podoplanin (21). Additionally, O-GalNAc glycans are enriched in neuronal tracts and participate in regulating the structure of nodes of Ranvier, thereby being associated with the neural transmission and axonal demyelination (22).

Gangliosides are indispensable for membrane organization, synaptogenesis and myelination. The complex gangliosides GM1, GD1a, GD1b and GT1b accumulate sharply during the perinatal and early postnatal period, and defects in their biosynthesis (deficiency of glycosyltransferase ST3GAL5, ST3GAL3 and B4GALNT1) are associated with early-onset epileptic encephalopathy (23).

Lastly, GPI anchors play a key role in sorting and membrane trafficking of several proteins. Defects in the GPI biosynthesis pathway (e.g., PIGW, PIGG, PIGO, and PGAP2) cause mislocalization of ion channels and neurotransmitter receptors. These deficiencies affect the metabolism of the vitamins as well as the production of energy by neurons, thus decreasing the production of inhibitory neurotransmitters (24). This cascade of molecular dysfunction to circuit level instability promotes the formation of abnormal neural networks and persistent epileptogenic foci.

The brain glycome is not static but follows a developmental timeline, which may help explain the differing ages of epilepsy onset across CDG subtypes. During the embryonic period, high-mannose and oligomannose N-glycans and polysialylated NCAM predominate, supporting neural-tube patterning, proliferation and migration; defects acting at these early steps (e.g., PMM2-, ALG- and DPAGT1-CDG) tend to manifest with cortical malformations and early onset epileptic encephalopathy. From the perinatal period through infancy, N-glycans become more complex and highly branched, with preferential sialylation occurring on the branches; the variety of gangliosides increases, accompanied by a peak in synapse formation, maturation of the GABAergic system, and rapid progression of myelination (25, 26); defects affecting these processes (e.g., SLC35A2, the GPI-anchor disorders and ganglioside-synthesis defects) more often present with focal seizures, infantile spasms or later-onset epileptic encephalopathy. Therefore, the effects of glycosylation defects at different stages of brain development may influence the onset of epilepsy, seizure characteristics, and associated conditions, although the relationship between different glycosylation subtypes and clinical phenotypes has yet to be established.

### 2.2 CDG and pediatric epilepsy

As mentioned above, epilepsy occurring in early childhood is a common clinical manifestation of CDG. Although more than 190 CDG types are now recognized, the epileptic features of only a few subtypes have been systematically summarized, and comprehensive clinical data on the epileptic features of many rare CDGs remain lacking. Table 1 summarizes the clinical characteristics of several representative CDG-associated epilepsies, including the seizure types, typical age of onset, and reported drug responsiveness. It should be noted that most of the evidence for these subtypes comes from small case series or individual case reports, and there may be reporting bias.

**Table 1:** CDG subtypes associated with epilepsy: comparison table of seizure types, age of onset, and antiepileptic treatment.

CDG subtype	Predominant seizure type(s)	Typical age of onset	Drug responsiveness/notes
*PMM2-CDG* (7)	Focal and generalized; stroke-like episodes	Infancy to early childhood	Generally good control; levetiracetam often effective, benzodiazepines for stroke-like episodes, 6.9% drug-resistant
*ALG13-CDG* (66)	Spasms; tonic; myoclonic; atonic, focal seizures;	Infancy	Spasms respond to ACTH/prednisolone or vigabatrin but with high recurrence rate
*ALG6-CDG* (8)	Myoclonic; spasms; tonic–clonic; focal seizures	Infancy	About 22% drug-resistant
*DPAGT1-CDG* (67)	Spasms, multifocal	Early infancy	Often drug-resistant; severe psychomotor retardation
*SLC35A2-CDG* (68)	Focal seizures, spasms	Infancy	Poor drug response; Galactose supplementation may help; surgery effective in MOGHE
*ST3GAL5-CDG* (69)	Myoclonic, tonic, tonic–clonic, focal seizures	Infancy	Poor drug response
*DHDDS-CDG* (70)	Myoclonic (often presenting with eyelid myoclonus); tonic–clonic, absence, atonic/myoclonic-atonic seizures, status epilepticus	Infancy to late adulthood	Drug responses vary, valproic acid tends to be effective
*RFT1-CDG* (71, 72)	Myoclonus, migrating partial/focal seizures	Infancy	Poor drug response, often associated with sensorineural hearing loss
*NGLY1-CDG* (73)	Myoclonic, atonic, focal, spasms, Tonic, absence, gelastic seizures	Late Infancy to adolescence	About one-third were able to achieve complete seizure control
GPI-anchor deficiencies (e.g., PIGA, PIGN, PIGO) (74)	Fever sensitivity, spasms, tonic, Myoclonic, focal, atypical absence seizure	Neonatal to infancy	Frequently refractory; pyridoxine and the ketogenic diet beneficial in some

### 2.3 Glycosylation disorders disrupt neural excitation-inhibition balance

In addition to its effects on early neurodevelopment, glycosylation abnormalities contribute to the initiation and maintenance of epilepsy by multiple pathological mechanisms. These multifaceted mechanisms lead to alteration of electrophysiological properties, synaptic dysfunction and dysregulated apoptotic signaling, which eventually results in destabilization of the brain’s critical excitation-inhibition balance.

#### 2.3.1 Voltage-gated ion channels

In the healthy brain, the activation and inactivation of voltage-gated ion channels are carefully regulated. Negatively charged N-linked glycans, often sialylated, are generally abundant on the extracellular surface of channel proteins. These sugar chains serve as key regulators, greatly modifying both the firing frequencies of axons and overall channel kinetics. Voltage-gated sodium channels (Nav) are essential for the generation of action potentials, and mutations affecting their function are often linked to hereditary epilepsies. N-glycosylation plays a significant role in the gating of various Nav subtypes, such as Nav1.3, Nav1.4, Nav1.5, Nav1.6, and Nav1.9. Changes at these sites directly affect activation and inactivation processes that are voltage dependent (3, 13, 27).

The correct glycosylation is also crucial for the functional integrity of potassium channels (Kv). For instance, abnormal glycosylation of Kv3.3 markedly reduces its level on the cell membrane, slowing down repolarization and increasing seizure susceptibility, which is also part of the spinocerebellar ataxia type 13 (27) pathology. In addition, there are several other subtypes such as Kv1.1–1.5, Kv1.7, Kv3.1 and Kv12.2, that have key glycosylation sites. Stability, trafficking efficiency and gating kinetics of these proteins depend on the glycosylation status (3).

N-glycosylation also regulates the surface expression and gating of the voltage-gated calcium channels Cav1.2 and Cav3.2; in particular, for Cav3.2, it underlies the glucose-dependent enhancement of channel transport to the membrane (28). In a similar fashion, the hyperpolarization-activated cyclic nucleotide-gated channels that mediate the hyperpolarization-activated *I_h_* current are heavily glycosylated. The extent of this modification dictates the density of functional channels on the cell membrane, so that the excitability of neurons and networks can be calibrated precisely (29).

#### 2.3.2 Neurotransmitter receptors and synaptic function

GABAₐ receptors are strongly regulated by N-linked glycosylation, whereas the excitatory receptors AMPA and NMDA are regulated by both N-glycosylation and O-glycosylation; defects in glycosylation can comprehensively affect subunit assembly, endoplasmic reticulum quality control, ligand affinity, and channel opening kinetics (30–32). In addition, subcellular targeting and synaptic localization of many vesicle proteins depends on N-glycosylation, which is essential for the regeneration and maintenance of synaptic vesicle pools (33). Many other key synaptic proteins are also sialylated glycoproteins, including subunits of the vacuolar ATPase (V-ATPase), synaptophysin, synaptoporin, and neurotransmitter transporters Slc6a17 and Slc24a2 (13). Together these glycosylated molecules coordinate the synaptic vesicle cycle and the efficiency of neurotransmitter release. Disruptions of glycosylation at the molecular level thus ultimately lead to the pathological dynamic remodeling seen at the neural network level in epilepsy.

#### 2.3.3 Neuroinflammation and neuronal death

Endoplasmic reticulum (ER) stress is the major pathway by which glycosylation defects trigger neuronal damage. Early-stage N-linked glycosylation is a key mediator of the unfolded protein response (UPR). In this pathway, specific glycosylated peptides are recognized by the chaperone protein calnexin, which helps the proteins refold. If the amount of misfolded proteins generated by a glycosylation defect exceeds the compensating power of the UPR, the resulting ER stress, redox imbalance and calcium toxicity inevitably result in neuronal apoptosis (2).

N-linked glycosylation is also extensively involved in neuroinflammatory pathways such as NF-κB, MAPK, and JAK/STAT, while cytokines and their receptors (e.g., IL-6, IFN-*γ*), inducible nitric oxide synthase (iNOS), and complement C3 are themselves glycoproteins whose N-glycosylation governs receptor–ligand binding, trafficking, and release (2). Lectin receptors on astrocytes and microglia (including galectins, sialic acid-binding Siglecs, and mannose-binding lectins) read glycan signals to establish the balance between pro-inflammatory and anti-inflammatory states: Siglecs recognize terminal sialic acid to maintain a resting, anti-inflammatory state, whereas surface desialylation promotes microglial activation and phagocytosis (34). On the other hand, in pathologies such as epilepsy, disruption of the blood–brain barrier allows platelets to enter the central nervous system, where they amplify neuroinflammation via the S100B-TLR-NF-κB/MAPK axis, release serotonin, increase barrier permeability, and stimulate neuronal discharge (35).

Moreover, glycosylation plays a crucial role in regulating the phagocytic function and neurotoxic effects of microglial cells, including modulation of the NF-κB, MAPK and JAK/STAT pathways and nitric oxide synthase (NOS) and C3 complement activity. This immunological balance is disturbed by pathological glycosylation patterns which lead to chronic activation of microglia and astrocytes. Prolonged neuroinflammation results in overproduction of reactive oxygen species and pro-apoptotic factors that in turn cause extensive and irreversible damage to neurons (2).

### 2.4 Glycosylation changes in epileptic brains trigger dynamic remodeling of neural networks

Genetically determined glycosylation defects are the upstream cause of a lowered seizure threshold, while recurrent seizures may induce glycosylation changes that exacerbate network instability. On this basis, we propose—as a working hypothesis—that these two processes may constitute a self-reinforcing (“vicious”) cycle, in which the primary defect and the seizure-induced changes mutually reinforce each other and drive disease progression. In hippocampal tissue resected from patients with refractory TLE, reduced levels of OGT were observed in the epileptogenic hippocampal tissue, accompanied by a significant decrease in overall O-GlcNAcylation levels (36). A high-resolution tandem mass spectrometry study of the human TLE-specific gangliosidome profile revealed that ganglioside expression was different in TLE, particularly with respect to the sialylation degree of components (37). Because these observations derive from chronically epileptic, surgically resected tissue, they cannot by themselves establish whether the glycomic changes are a cause, a consequence, or a compensatory response to recurrent seizures, and this directionality remains a hypothesis requiring prospective validation. That experimentally restoring O-GlcNAcylation suppresses epileptiform activity, however, indicates that at least part of this secondary change is functionally contributory rather than epiphenomenal (38). These glycosylation changes may lead to the remodeling of neural networks by regulating ion channel gating, receptor surface expression, synaptic plasticity, and axonal outgrowth, contributing to the chronicity of epilepsy and may even trigger status epilepticus. Distinguishing and jointly managing the pathway remodeling of primary congenital defects and secondary epilepsy has practical significance: congenital glycosylation defects can be corrected through substrate supplementation and gene-targeted therapies, whereas secondary remodeling triggered by epileptic seizures is primarily addressed by effectively controlling seizures and improving energy metabolism pathways.

### 2.5 Abnormal glycosylation participates in the drug-resistant epilepsy

**Figure 1:**
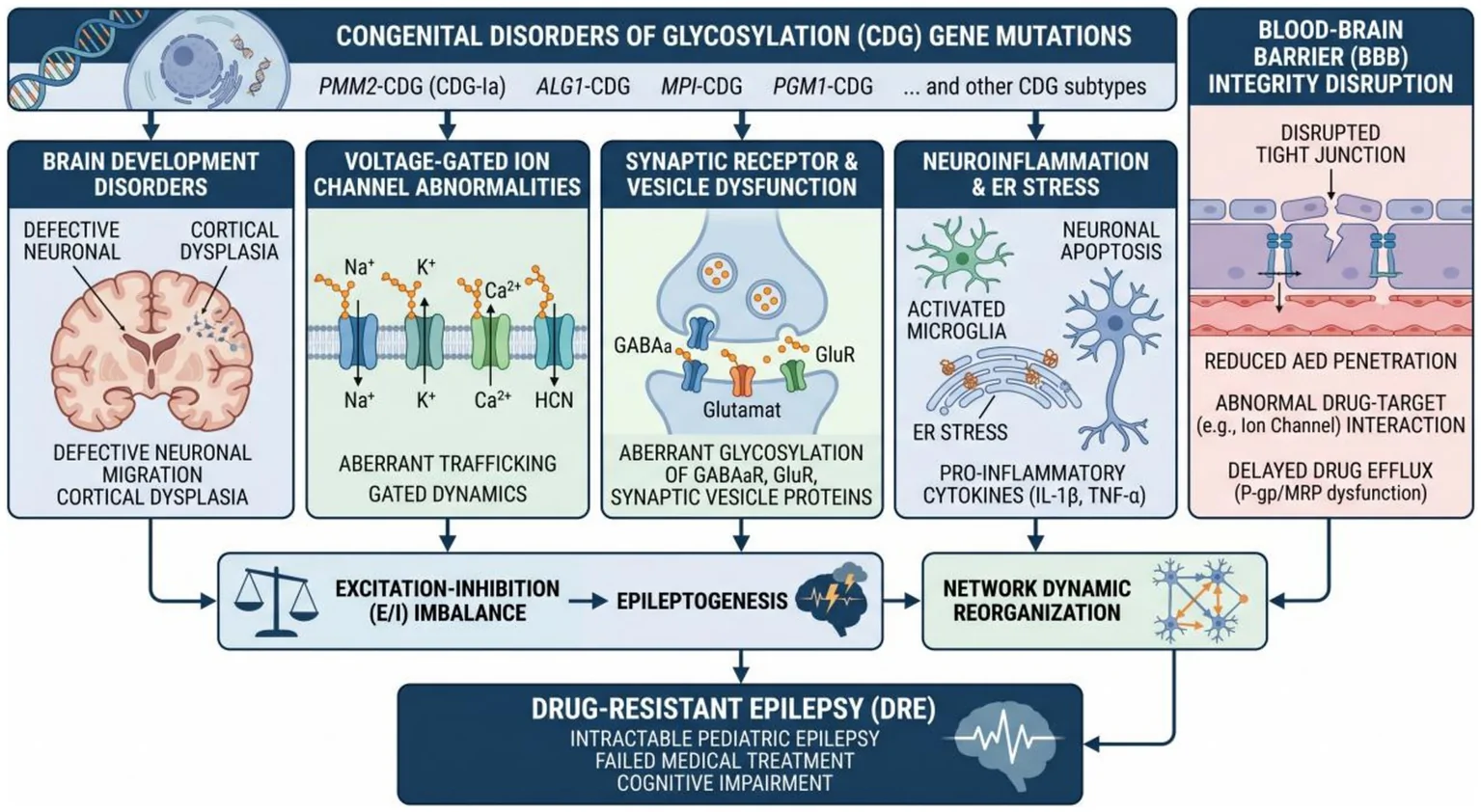
Multimodal pathogenic mechanisms underlying drug-resistant epilepsy in congenital disorders of glycosylation.

There is increasing evidence that a combination of neuroinflammation, altered immune responses, and blood–brain barrier (BBB) breakdown is associated with the development of drug-resistant epilepsy (39). Since glycosylation is an intrinsic part of these pathophysiological processes, disruption of glycosylation is likely a major trigger of pharmacoresistance. This impact is most evident at the molecular level, as most of the targets of the primary antiepileptic drugs (AEDs) – including ion channels and neurotransmitter receptors – are glycoproteins. Pathological glycosylation can affect the conformational and functional properties of these proteins, reduce affinity for drugs or interfere with important signal transduction pathways, resulting in therapeutic failure (40). For example, in classic Nav-blocking AEDs, carbamazepine, phenytoin, and lamotrigine preferentially bind to and stabilize the fast-inactivated state, whereas glycosylation participates in regulating the voltage dependence of Nav’s steady-state inactivation and rearranges its conformational distribution (41). Thus, glycosylation indirectly influences the accessibility of these AED-binding conformations. Moreover, glycosylation plays an important role in the pharmacokinetics of AEDs. Examples include, in the case of *UGT1A1*, defects that lower the glycosylation of valproate slow its clearance and, in other glycosylation disorders, the upregulation of certain UDP-glucuronosyltransferases may increase the rate of drug metabolism and prevent attainment of therapeutic blood concentrations (42). Key BBB components such as P-glycoprotein and multidrug resistance-associated proteins are also glycosylated. The function of the BBB may be compromised through abnormal modification of these transporters, which also appears to increase the clearance of drugs and thus further enhance resistance (43). Finally, the involvement of advanced glycation end products (AGEs), which are non-enzymatically glycated proteins, lipids or nucleic acids, complicates the neuroinflammatory landscape by activation of specific AGE receptors (RAGE). Prolonged activation of AGE/RAGE axis not only promotes the exacerbation of neuronal injury but also creates a chronic inflammatory microenvironment, which may trigger drug resistance (44). At present, however, no controlled study has directly compared AED responsiveness between patients with and without CDG; the literature is limited to subtype-specific case series, and head-to-head pharmacological comparisons remain an important and unmet research need. Figure 1 shows the various disease mechanisms that can be attributed to abnormal glycosylation and drug-resistant epilepsy.

## 3 Neuroimaging features of glycosylation disorder-related epilepsy

The neuroimaging features of glycosylation disorders are very variable and include many structural developmental abnormalities. Common abnormalities noted are cortical malformations including lissencephaly, pachygyria, polymicrogyria and neuronal heterotopia and midline abnormalities including corpus callosum malformation and pontocerebellar hypoplasia. In addition, patients frequently have abnormalities in brain volume such as enlargement of the ventricles, hydrocephalus, and progressive and non-progressive global and cerebellar atrophy, as well as abnormalities in myelination and cerebrovascular abnormalities (45). Table 2 lists some reported cranial MRI manifestations of CDG.

**Table 2:** Common neuroimaging abnormalities in CDG-associated epilepsy.

Imaging abnormality	Associated CDG subtypes
Brain atrophy	*PMM2, SLC35A2, ALG11, DOLK, ALG13* (15)*, SEC24* (75), *PIGG, PIGA, PIGL, PIGV, PIGN, PIGO, PIGT, PGAP1/3* (74)*, CAD* (76), *MOGS* (77)*, ALG12* (78), *COG* (15), *UGDH* (79)*, NANS* (80)
Cerebellar atrophy	*PMM2, SLC35A2, B3GALNT2, DPM2, ALG13* (15), *SEC24* (75), *PIGG, PIGA, PIGL, PIGV, PIGN, PIGO, PIGT, PGAP1/3* (74)*, CAD* (76), *ALG12* (78), *GALNT2* (81), *GPAA1* (82), *UGGT1* (83), *TRAPPC9* (84), *GMPPB* (85), *NUS1* (86), *UGDH* (79), *DHDDS* (87), *SRD5A3* (88), *FCSK* (89), *NANS* (80), *RPN1* (90)
Ventriculomegaly	*SLC35A2* (91)*, B3GALNT2, FKTN, ISPD, NANS, POMGNT1, POMT2, FKRP, ALG12, ALG13* (15), *SEC24* (75), *COG* (92), *PIGA, PIGL, PIGT, PGAP3* (74), *MOGS* (77), *ALG11* (93), *UGDH* (79)
Myelination/white matter abnormalities	*TMEM165* (94), *B3GALNT2, DOLK, PGM1, DPM2, ALG2/9/13, DDOST, DPM1, SLC35A2* (15), *SEC24* (75), *GALNT2* (81), *COG* (95), *UGGT1* (83), *TRAPPC9* (84), *PIGA, PIGL, PIGV, PIGT, PGAP1/3* (74), *FCSK* (89)
Cortical dysplasia	*SLC35A2, B3GALNT2, FKRP, FKTN, ISPD, POMT1/2, POMGNT1* (15), *UGGT1* (83)
Corpus callosum dysplasia	*SEC24* (75), *FKRP, FKTN, NANS, POMGNT1, POMT1/2, SLC35A2, COG, PMM2* (15), *ALG13* (52), *SSR4* (96), *PIGO, PIGG, PIGA, PIGL, PIGT, PGAP3* (74), *UGGT1* (83), *TRAPPC9* (84), *FCSK* (89), *RPN1* (90), *NANS* (80)

Although most individual features are non-specific, certain patterns can narrow the differential toward particular subtypes. Olivopontocerebellar hypoplasia or progressive cerebellar atrophy with a hypoplastic vermis should prompt consideration of PMM2-CDG (7); a cobblestone cortical malformation with cerebellar cysts and brainstem hypoplasia points to the dystroglycanopathies (46); and a focal frontal lesion with blurring of the grey–white matter junction and subcortical oligodendroglial hyperplasia is the radiological signature of SLC35A2-related MOGHE (47).

These imaging findings have an identifiable pathological basis. The characteristic olivopontocerebellar atrophy in PMM2-CDG reflects the loss of cerebellar Purkinje cells and granule cells driven by hypoglycosylation. Cerebellar granule cells are more sensitive to N-glycosylation defects than cortical neurons because these neurons exhibit an inadequate compensatory response to endoplasmic reticulum (ER) stress induced by N-glycosylation defects, leading to progressive atrophy after birth (48). By contrast, *α*-dystroglycan is localized to radial glial end-feet, and its glycosylation is essential for maintaining the integrity of the glia limitans-basement membrane complex. Defects in O-mannosylation (POMT1/2, FKTN, FKRP, etc.) result in hypoglycosylation of α-dystroglycan, causing it to lose its ability to bind to basement membrane. Neurons then over-migrate into the subpial/subarachnoid spaces, leading to cobblestone lissencephaly (49). Loss-of-function SLC35A2 in brain somatic mosaicism reduces the supply of galactose to the Golgi lumen, leading to global hypogalactosylation of N-glycans and sphingoglycolipids, disrupts axon-oligodendrocyte adhesion, and results in impaired neuronal migration, which pathologically manifests as patchy hypomyelination, oligodendroglial hyperplasia, and cortical ectopia (47).

CDG-associated imaging abnormalities may be static or progressive, and this distinction is useful for diagnosis. Migration defects and other cortical dysgenesis are fixed, reflecting a discrete prenatal injury, whereas atrophic changes—such as cerebellar atrophy in PMM2-CDG and cerebral atrophy in ALG11-CDG—are typically progressive. They may not be radiologically apparent during the fetal period or early infancy and become evident only on serial imaging. Therefore, serial MRI is valuable for both predicting and distinguishing between progressive metabolic processes and static structural lesions. It is important to note that no single imaging finding is diagnostic; its primary value lies in raising suspicion of a glycosylation disorder, guiding the selection of confirmatory testing, and tracking the disease’s progression, rather than establishing a subtype-specific diagnosis on its own.

## 4 From clinical suspicion to diagnosis: a stepwise pathway and biomarker panel

Because the seizure phenotypes and imaging features described above are individually non-specific, a structured, tiered work-up is required to move from clinical suspicion to a molecular diagnosis. We propose the following pragmatic pathway, which integrates the clinical, biochemical, genetic and imaging information discussed in the preceding sections and is intended to be rendered as a clinical algorithm.

Step 1—When to suspect a glycosylation disorder. CDG should be considered in any infant or child with early-onset or otherwise unexplained epilepsy accompanied by one or more “red-flag” features: global developmental delay or regression, axial hypotonia, failure to thrive, dysmorphism (inverted nipples and abnormal subcutaneous fat distribution are classic for PMM2-CDG), strabismus, cerebellar signs or ataxia, and multisystem involvement such as hepatopathy, coagulopathy (reduced antithrombin, factor XI, or protein C/S), protein-losing enteropathy, and cardiac or endocrine abnormalities. Drug-resistant seizures without a clear acquired cause, or a suggestive MRI pattern (Section 3), should further raise the index of suspicion.

Step 2—First-line screening test for laboratories with capabilities, serum transferrin isoform analysis and apolipoprotein C-III isoform analysis can serve as screening tests for most N-glycosylation and mucin O-glycosylation defects, while liquid chromatography and matrix-assisted laser desorption/ionization time-of-flight mass spectrometry can be used to analyze the complete structure, chain length, and composition of glycans (50). Crucially, other O-glycosylation defects (such as O-mannosylation and O-GlcNAc), GPI anchoring disorders, ganglioside/sphingolipid synthesis defects, and dystroglycanopathies cannot be detected by the above screening methods, so screening must always be interpreted together with the clinical picture. Concurrent routine investigations (liver enzymes, a coagulation panel and thyroid function) help to map the extent of multisystem involvement.

Step 3—Confirmatory targeted CDG/epilepsy gene testing or, preferably, trio whole-exome or whole-genome sequencing — is the definitive diagnostic step and should be pursued whenever clinical suspicion is high, even when screening is normal. For SLC35A2-associated MOGHE, deep sequencing of the resected epileptogenic tissue may be required.

Step 4—If a variant of unknown significance (VUS) is detected, the following methods can be used to verify its pathogenicity: targeted enzyme assays (e.g., testing for phosphomannomutase and phosphomannose-isomerase activity when PMM2-CDG or MPI-CDG is suspected), analysis of lipid-linked oligosaccharides, and mass-spectrometry glycomic profiling of serum N- and O-glycans. For GPI anchoring defects, flow cytometry can be used to detect GPI-anchored proteins on the surface of granulocytes (such as FLAER and CD16), thereby confirming the pathogenicity of genetic variants at the functional level.

Step 5—Neuroimaging (e.g., MRI) is used to identify subtypes and monitor changes over time; for example, olivopontocerebellar hypoplasia suggests PMM2-CDG, while a cobblestone cortical malformation suggests dystroglycanopathies (Section 3). Serial imaging can distinguish between static malformations and progressive atrophy and provide insights into prognosis.

Currently, no single biomarker or biomarker panel is available to aid in the sensitive and specific diagnosis of glycosylation-related epilepsy. Several promising markers remain at the research stage and are not yet validated for clinical use: cerebrospinal fluid and serum N-/O-glycan candidate markers (referencing changes in brain glycomics in neurodegenerative diseases), ganglioside sialylation profiles (temporal lobe epilepsy gangliosome), and O-GlcNAc levels in tissues (such as the epileptogenic hippocampus) (4, 36, 37). To incorporate these candidate biomarkers into clinical diagnostic workflows, it is imperative to conduct multicenter prospective studies to validate their association with seizure prognosis and treatment response.

## 5 Treatment of glycosylation disorder-related epilepsy

Treatment of CDG requires a very individualized approach and the semiology of the seizures can be very varied depending on the genetic subtype. Levetiracetam monotherapy often works well for *PMM2-CDG* but benzodiazepines are the treatment of choice for stroke-related episodes (7, 51). Approximately 87% of patients with ALG13-CDG show an initial response to treatment with ACTH or prednisolone; however, the relapse rate is high, and most patients experience a recurrence of seizures after the dose is reduced or the medication is discontinued. Clobazam, felbamate and vigabatrin can be used as adjuncts for more general seizures (52). Valproate is often used for myoclonic seizures, and levetiracetam, brivaracetam, and clonazepam are also options (45, 53). In DHDDS-CDG, seizures are mostly myoclonic and other seizures may include generalized tonic–clonic, absence, atonic and status epilepticus; valproic acid is most effective for seizures, and levetiracetam, lamotrigine, etc., are variable (12). In addition, therapeutic breakthroughs have led to the use of cannabidiol as a powerful treatment to decrease the frequency of seizures in *ALG11*-CDG (54). In addition to pharmacological treatments, the ketogenic diet has proven to be a revolutionary non-targeted therapy, providing a remarkable reduction in seizures and sometimes complete remission for patients with GPI anchor protein deficiency or *SLC35A2, ALG13,* and *ALG3* mutations (15, 45, 55).

The mechanisms by which the ketogenic diet benefits glycosylation disorders are likely to be multimodal and extend beyond simple seizure suppression. First, ketone bodies (*β*-hydroxybutyrate and acetoacetate) provide an alternative oxidizable substrate that can stabilize neuronal energy (56). Second, the hexosamine-biosynthetic pathway branches from glycolysis and generates UDP GlcNAc, which serves as an O-GlcNAc donor and acts as a glucose-dependent nutrient sensor (2, 57); therefore, reducing glucose utilization may theoretically regulate UDP-GlcNAc and O-GlcNAcylation. Third, *β*-hydroxybutyrate is an endogenous HDAC inhibitor and signaling molecule that influences gene expression and inhibits neuroinflammatory pathways such as NLRP3 (58).

Safety, however, requires particular caution in CDG. Many subtypes—most notably PMM2-CDG, ALG8-CDG and PGM1-CDG—feature intrinsic hepatic involvement, coagulopathy and hypoglycaemia, all of which the high-fat, carbohydrate-restricted ketogenic diet can aggravate. In patients with pre-existing liver dysfunction the diet should be undertaken with close hepatic and metabolic monitoring, attention to coagulation status, and avoidance of prolonged fasting (59–61). Thus the ketogenic diet should be individually tailored for each patient, with its potential antiseizure benefit weighed against hepatic and metabolic risk. After initiating a ketogenic diet, blood glucose, blood ketones, liver function, coagulation, and growth and development indicators must be closely monitored (62).

Targeted therapies in the glycosylation pathway are a new and exciting field for precision medicine in CDG related epilepsy. A number of experimental studies indicate that the O-GlcNAcase (OGA) is a potential therapeutic target: for example, the OGA inhibitor thiamet-G was reported to reduce the duration of seizures and decrease spike activity in rats with temporal lobe epilepsy by increasing protein O-GlcNAcylation (36). In cases where *SLC35A2* is involved (including MOGHE), surgical resection of the epileptogenic focus is still a very effective treatment for structural anomalies such as focal cortical malformations (18). Although preclinical and clinical data suggest the potential efficacy of a number of emerging agents (shown in Table 3), further optimization is needed to improve their safety and specificity characteristics. Moreover, substrate supplementation therapy is frequently used on a compassionate or off-label basis and is an important proof of concept for “metabolic reprogramming.” While only effective in certain groups of CDG patients, its potential of metabolic correction is a major paradigm change for precision-based neurological care.

**Table 3:** Targeted therapeutic strategies for glycosylation disorder–related epilepsies.

Therapeutic category	Agent	CDG subtypes/epilepsy
Substrate supplementation	Galactose	*PGM1, ALG13, PMM2* (97)*, SLC35A2, SLC39A8, TMEM165* (98)*, GNE* (54)
	Mannose	*MPI* (99)
	Fucose	*SLC35C1* (98)*, FUT8, GFUS* (55)
	Uridine	*CAD* (98), PGM*1, DPAGT1, GNE* (54)
	Pyridoxine, vitamin B1, B2, folate	*SLC35A2* (15)*, PMM2* (100), GPI deficiencies (*PGAP3, PIGN, PIGA, PIGL, PIGV, PIGO* and *PIGM-CDG*) (55)
	N-acetylglucosamine	*PGM1, GNE* (54)
	Manganese	*SLC39A8, TMEM165* (55)
	Sodium Butyrate	*PIGM* (55)
	Sialic acid	*GNE* (87)
	N-acetylmannosamine	*GNE, MPI* (87)
	Metformin	*PMM2* (101)
Chemically synthesized intermediates	Liposome-encapsulated mannose-1-phosphate	*PMM2* (102)
	GlcNAc-PI/GlcN-PI	GPI deficiency (24)
Enzyme inhibitors	Aldose reductase inhibitors (epalrestat, etc.)	*PMM2* (54)
	Histone deacetylation inhibitors	*PIGM* (103)
	Benzoisothiazolone compounds	*PMM2* (104)
	Acetazolamide	*PMM2* (104)*, DHDDS* (105)
Pharmacological chaperones	Celastrol	*PMM2* (104)
ER stress inhibitors	Salubrinal	*PMM2, DPAGT1, NGLY1* (101)
Gene therapy	AAV vectors	*GNE* (98)*, PGM1* (54)*, FKRP* (54)*, NGLY1* (101)*, PMM2* (87)
	Antisense oligonucleotide Therapy	*TMEM165* (98)*, PMM2* (104)

Level of evidence (LoE). The strategies listed differ markedly in the strength of supporting evidence. We apply three tiers: LoE A, human clinical data (case series or case reports, including off-label or compassionate use); LoE B, preclinical in-vivo data (animal models); and LoE C, in-vitro or biochemical proof-of-concept only. By category: substrate supplementation (galactose, mannose, fucose, uridine, manganese, vitamins, N-acetylglucosamine, sialic acid) rests predominantly on LoE A case series, strongest for mannose in MPI-CDG and galactose in PGM1- and SLC35A2-CDG; acetazolamide (PMM2-, DHDDS-CDG) and metformin (PMM2-CDG) are also LoE A. Enzyme inhibitors (aldose-reductase inhibitors such as epalrestat, HDAC inhibitors, benzoisothiazolones), pharmacological chaperones (celastrol) and ER-stress inhibitors (salubrinal) are largely LoE C with emerging LoE B support. Gene-replacement (AAV) and antisense approaches are currently LoE B or LoE C and have not yet reached registered clinical trials. Readers are referred to the per-agent citations for the specific evidence base.

Gene-directed therapies represent the most forward-looking strand of CDG therapeutics. For PMM2-CDG, adeno-associated virus (AAV)-mediated gene replacement has advanced through proof-of-concept and animal studies: AAV9-PMM2 delivery restored PMM2 expression and improved glycosylation in patient-derived fibroblasts, and in a tamoxifen-inducible Pmm2-knockout mouse model AAV9-PMM2 both prevented and halted the emergence of disease-relevant neurological phenotypes (63). These data establish a rationale for clinical translation, although no AAV gene-replacement product for PMM2-CDG has yet entered registered clinical trials, and durability, vector tropism to the central nervous system and immunogenicity remain to be addressed. Antisense oligonucleotide (ASO) therapy corrects splicing abnormalities by forcing the skipping of pseudo-exons, thereby restoring normal transcripts and proteins. In PMM2-CDG, co-transfection of two morpholino oligomers restored PMM enzyme activity in patients’ fibroblasts to approximately 50% of that in healthy controls after 48–72 h (64). In TMEM165-CDG, an antisense morpholino directed against a deep-intronic variant (c.792 + 182G > A) recovered normal protein levels in the Golgi apparatus of patient-derived fibroblasts (65). Both modalities exemplify the shift from symptomatic seizure control toward correction of the underlying glycosylation defect.

## 6 Conclusion

Among post-translational modifications in childhood epilepsy, glycosylation is emerging as a key area of research, rather than being regarded merely as a rare cause of metabolic epilepsy. In the developing brain, glycosylation regulates neuronal migration, ion channel and receptor function, synaptic transmission, and neuroimmune homeostasis; consequently, glycosylation defects affect the excitatory–inhibitory balance of neural circuits across multiple dimensions. Beyond this primary role, existing but still limited data also suggest that this relationship may be bidirectional—that is, recurrent seizures may in turn remodel the brain glycome, contributing to the development of chronic and drug-resistant epilepsy. This pathophysiological framework has clear clinical implications: in children with early-onset, multisystem, drug-resistant epilepsy—particularly those with progressive brain atrophy—glycosylation abnormalities should be included as a key screening target; at the same time, further evidence on secondary, seizure-related glycosylation changes is needed to identify new therapeutic targets and to reduce chronicity and drug resistance. Accordingly, priorities for future work include validating glycans, O-GlcNAc, and related molecules as biomarkers and establishing their value as intervention targets through multicenter, large-scale prospective pediatric cohorts, and elucidating the spatiotemporal dynamics of brain glycosylation by integrating multi-omics technologies with high-resolution neuroimaging. Furthermore, we recommend accelerating clinical trials of glycosylation-targeted, disease-modifying therapies in pediatric populations, with a focus on the challenges of central nervous system delivery efficiency, sustained efficacy, and pediatric-specific safety.

## References

[1] Yüksel MF Doğulu N Yıldırım M Köse E Bektaş Ö Eminoğlu FT . Metabolic etiologies in children with infantile epileptic spasm syndrome: experience at a tertiary pediatric neurology center. Brain Dev. (2024) 46:213–8. doi: 10.1016/j.braindev.2024.03.003, 38493042

[2] Conroy LR Hawkinson TR Young LEA Gentry MS Sun RC. Emerging roles of N-linked glycosylation in brain physiology and disorders. Trends Endocrinol Metab. (2021) 32:980–93. doi: 10.1016/j.tem.2021.09.006, 34756776 PMC8589112

[3] Hilary S Panin VMJG. The role of protein N-glycosylation in neural transmission. Glycobiology. (2014) 5:407. doi: 10.1093/glycob/cwu015 PMC3976283 24643084

[4] Tena J Lebrilla CB. Glycomic profiling and the mammalian brain. Proc Natl Acad Sci USA. (2021) 118:e2022238118. doi: 10.1073/pnas.2022238118, 33380465 PMC7817126

[5] Ng BG Freeze HH. Perspectives on glycosylation and its congenital disorders. Trends Genet. (2018) 34:466–76. doi: 10.1016/j.tig.2018.03.002, 29606283 PMC5959770

[6] Tumiene B Ferreira CR van Karnebeek CDM. 2022 Overview of metabolic epilepsies. Genes (Basel). (2022) 13:508. doi: 10.3390/genes13030508 35328062 PMC8952328

[7] Muthusamy K Perez-Ortiz JM Ligezka AN Altassan R Johnsen C Schultz MJ . Neurological manifestations in PMM2-congenital disorders of glycosylation (PMM2-CDG): insights into clinico-radiological characteristics, recommendations for follow-up, and future directions. Genet Med. (2024) 26:101027. doi: 10.1016/j.gim.2023.101027, 37955240 PMC13723945

[8] Morava E Tiemes V Thiel C Seta N de Lonlay P de Klerk H . ALG6-CDG: a recognizable phenotype with epilepsy, proximal muscle weakness, ataxia and behavioral and limb anomalies. J Inherit Metab Dis. (2016) 39:713–23. doi: 10.1007/s10545-016-9945-x, 27287710

[9] Lipiński P Tylki-Szymańska A. Congenital disorders of glycosylation: what clinicians need to know? Front Pediatr. (2021) 9:715151. doi: 10.3389/fped.2021.715151 34540767 PMC8446601

[10] Yu H Chen X Yang Y Gu M Ren K Wei Z. Decoding glycosylation in neurodegenerative diseases: mechanistic insights and therapeutic opportunities. FASEB J. (2025) 39:e71160. doi: 10.1096/fj.202502809R, 41110100 PMC12535759

[11] Williams SE Mealer RG Scolnick EM Smoller JW Cummings RDJMP. Aberrant glycosylation in schizophrenia: a review of 25 years of post-mortem brain studies. Mol Psychiatry. (2020) 25:3198–3207. doi: 10.1038/s41380-020-0761-1 32404945 PMC8081047

[12] Lv T Fu JX Liu XY Tang R Yang GL. Case analysis of epilepsy, neurodevelopmental disorder, and motor disorders associated with mutations in the dehydrodolichyl diphosphate synthase gene. Seizure. (2023) 110:126–35. doi: 10.1016/j.seizure.2023.06.006, 37356182

[13] Boll I Jensen P Schwämmle V Larsen MR. Depolarization-dependent induction of site-specific changes in sialylation on N-linked glycoproteins in rat nerve terminals. Mol Cell Proteomics. (2020) 19:1418–35. doi: 10.1074/mcp.RA119.001896, 32518069 PMC8143646

[14] Mehrabian M Hildebrandt H Schmitt-Ulms G. NCAM1 polysialylation: the prion protein's elusive reason for being? ASN Neuro. (2016) 8:1–25. doi: 10.1177/1759091416679074, 27879349 PMC5122176

[15] Paprocka J Jezela-Stanek A Tylki-Szymańska A Grunewald S. Congenital disorders of glycosylation from a neurological perspective. Brain Sci. (2021) 11:88. doi: 10.3390/brainsci11010088, 33440761 PMC7827962

[16] Benner O Cast TP Minamide LS Lenninger Z Bamburg JR Chanda S. Multiple N-linked glycosylation sites critically modulate the synaptic abundance of neuroligin isoforms. J Biol Chem. (2023) 299:105361. doi: 10.1016/j.jbc.2023.105361, 37865312 PMC10679506

[17] Sengupta PK Bouchie MP Nita-Lazar M Yang H-Y Kukuruzinska MA. Coordinate regulation of N-glycosylation gene DPAGT1, canonical Wnt signaling and E-cadherin adhesion. J Cell Sci. (2013) 126:484–96. doi: 10.1242/jcs.113035, 23178939 PMC3613180

[18] Liu XY Tang Q Xia XQ Liu QZ Liu JL Jin YYA . Somatic variants in SLC35A2 leading to defects in N-glycosylation in mild malformation of cortical development with oligodendroglial hyperplasia in epilepsy (MOGHE). Acta Neuropathol. (2025) 149:13. doi: 10.1007/s00401-025-02850-1, 39900685

[19] Schjoldager KT Narimatsu Y Joshi HJ Clausen H. Global view of human protein glycosylation pathways and functions. Nat Rev Mol Cell Biol. (2020) 21:729–49. doi: 10.1038/s41580-020-00294-x, 33087899

[20] Wenzel DM Olivier-Van Stichelen S. The O-GlcNAc cycling in neurodevelopment and associated diseases. Biochem Soc Trans. (2022) 50:1693–702. doi: 10.1042/bst20220539, 36383066 PMC10462390

[21] Yingjiao X Wenjie P Jishun L Aidong S Yan Z. Polypeptide N-Acetylgalactosaminyltransferase 13 contributes to neurogenesis via stabilizing the mucin-type O-glycoprotein podoplanin*. J Biol Chem. (2016) 291:23477–88. doi: 10.1074/jbc.M116.743955 27629416 PMC5095403

[22] Noel M Suttapitugsakul S Cummings RD Mealer RG. O-GalNAc glycans are enriched in neuronal tracts and regulate nodes of Ranvier. Proc Natl Acad Sci U S A. (2025) 122:e2418949122. doi: 10.1073/pnas.2418949122 39999163 PMC11892645

[23] Inamori K-i Inokuchi J-i. When ganglioside pathways go awry: congenital disorders and experimental insights. J Hum Genet. (2025). doi: 10.1038/s10038-025-01366-6, 40745477

[24] Murakami Y Kinoshita T. GPI anchor and its deficiency. Trends Glycosci Glycotechnol. (2024) 36:E1–5. doi: 10.4052/tigg.2331.1E

[25] Lee J Ha S Kim M Kim S-W Yun J Ozcan S . Spatial and temporal diversity of glycome expression in mammalian brain. Proc Natl Acad Sci U S A. (2020) 117:28743–53. doi: 10.1073/pnas.2014207117 33139572 PMC7682437

[26] Ngamukote S Yanagisawa M Ariga T Ando S Yu RK. Developmental changes of glycosphingolipids and expression of glycogenes in mouse brains. J Neurochem. (2007) 103:2327–41. doi: 10.1111/j.1471-4159.2007.04910.x 17883393

[27] Terragni B Scalmani P Franceschetti S Cestèle S Mantegazza M. Post-translational dysfunctions in channelopathies of the nervous system. Neuropharmacology. (2018) 132:31–42. doi: 10.1016/j.neuropharm.2017.05.028, 28571716

[28] Weiss N Zamponi GW. Trafficking of neuronal calcium channels. Neuronal Signal. (2017) 1:Ns20160003. doi: 10.1042/ns20160003, 32714572 PMC7373241

[29] Brennan GP Baram TZ Poolos NP. Hyperpolarization-activated cyclic nucleotide-gated (HCN) channels in epilepsy. Cold Spring Harb Perspect Med. (2016) 6:a022384. doi: 10.1101/cshperspect.a022384, 26931806 PMC4772079

[30] Lo WY Lagrange AH Hernandez CC Harrison R Dell A Haslam SM . Glycosylation of {beta}2 subunits regulates GABAA receptor biogenesis and channel gating. J Biol Chem. (2010) 285:31348–61. doi: 10.1074/jbc.M110.151449, 20639197 PMC2951209

[31] Tucholski J Simmons MS Pinner AL Haroutunian V McCullumsmith RE Meador-Woodruff JH. Abnormal N-linked glycosylation of cortical AMPA receptor subunits in schizophrenia. Schizophr Res. (2013) 146:177–83. doi: 10.1016/j.schres.2013.01.031, 23462048 PMC3655690

[32] Tucholski J Simmons MS Pinner AL McMillan LD Haroutunian V Meador-Woodruff JH. N-linked glycosylation of cortical N-methyl-D-aspartate and kainate receptor subunits in schizophrenia. Neuroreport. (2013) 24:688–91. doi: 10.1097/WNR.0b013e328363bd8a, 23820740 PMC3919653

[33] Kwon SE Chapman ER. Glycosylation is dispensable for sorting of synaptotagmin 1 but is critical for targeting of SV2 and synaptophysin to recycling synaptic vesicles. J Biol Chem. (2012) 287:35658–68. doi: 10.1074/jbc.M112.398883, 22908222 PMC3471705

[34] Läubli H Varki A. Sialic acid–binding immunoglobulin-like lectins (Siglecs) detect self-associated molecular patterns to regulate immune responses. Cell Mol Life Sci. (2020) 77:593–605. doi: 10.1007/s00018-019-03288-x, 31485715 PMC7942692

[35] Bo Y Zhao F. Platelets as central hubs of inflammation. Front Immunol. (2025) 16:1683553. doi: 10.3389/fimmu.2025.1683553 41103439 PMC12520916

[36] Sánchez RG Parrish RR Rich M Webb WM Lockhart RM Nakao K . Human and rodent temporal lobe epilepsy is characterized by changes in O-GlcNAc homeostasis that can be reversed to dampen epileptiform activity. Neurobiol Dis. (2019) 124:531–43. doi: 10.1016/j.nbd.2019.01.001, 30625365 PMC6379093

[37] Ica R Mlinac-Jerkovic K Ilic K Sajko T Munteanu CVA Zamfir AD . Gangliosidome of a human hippocampus in temporal lobe epilepsy resolved by high-resolution tandem mass spectrometry. Molecules. (2022) 27:4056. doi: 10.3390/molecules27134056, 35807302 PMC9268582

[38] Stewart LT Khan AU Wang K Pizarro D Pati S Buckingham SC . Acute increases in protein O-GlcNAcylation dampen epileptiform activity in Hippocampus. J Neurosci. (2017) 37:8207–15. doi: 10.1523/jneurosci.0173-16.2017, 28760863 PMC5566868

[39] Yu N Liu H Di Q. Modulation of immunity and the inflammatory response: a new target for treating drug-resistant epilepsy. Curr Neuropharmacol. (2013) 11:114–27. doi: 10.2174/157015913804999540, 23814544 PMC3580785

[40] Tanaka M Olsen RW Medina MT Schwartz E Alonso ME Duron RM . Hyperglycosylation and reduced GABA currents of mutated GABRB3 polypeptide in remitting childhood absence epilepsy. Am J Hum Genet. (2008) 82:1249–61. doi: 10.1016/j.ajhg.2008.04.020, 18514161 PMC2427288

[41] Isaev D Isaeva E Shatskih T Zhao Q Smits NC Shworak NW . Role of extracellular sialic acid in regulation of neuronal and network excitability in the rat hippocampus. J Neurosci. (2007) 27:11587–94. doi: 10.1523/jneurosci.2033-07.2007, 17959801 PMC6673228

[42] Dimunová D Matousková P Podlipná R Bousová I Skálová L. The role of UDP-glycosyltransferases in xenobioticresistance. Drug Metab Rev. (2022) 54:282–98. doi: 10.1080/03602532.2022.2083632, 35635097

[43] Kubota H Ishihara H Langmann T Schmitz G Stieger B Wieser HG . Distribution and functional activity of P-glycoprotein and multidrug resistance-associated proteins in human brain microvascular endothelial cells in hippocampal sclerosis. Epilepsy Res. (2006) 68:213–28. doi: 10.1016/j.eplepsyres.2005.11.011, 16361082

[44] Guo M Wang J Qi H Liu F Yao L Zhang S . Polymorphisms in the receptor for advanced glycation end products gene are associated with susceptibility to drug-resistant epilepsy. Neurosci Lett. (2016) 619:137–41. doi: 10.1016/j.neulet.2016.01.043, 26828298

[45] Lee HF Chi CS. Congenital disorders of glycosylation and infantile epilepsy. Epilepsy Behav. (2023) 142:109214. doi: 10.1016/j.yebeh.2023.109214, 37086590

[46] Clement E Mercuri E Godfrey C Smith J Robb S Kinali M . Brain involvement in muscular dystrophies with defective Dystroglycan glycosylation. Ann Neurol. (2008) 64:573–82. doi: 10.1002/ana.21482, 19067344

[47] Risso B Riva A Volpedo G Conti V di San Carlo CT Zara F . SLC35A2-related brain disorders: genetics, pathophysiology, and therapeutic insights. Int J Mol Sci. (2025) 26:11560. doi: 10.3390/ijms262311560, 41373710 PMC12692547

[48] Sun L Zhao Y Zhou K Freeze HH Zhang YW Xu H. Insufficient ER-stress response causes selective mouse cerebellar granule cell degeneration resembling that seen in congenital disorders of glycosylation. Mol Brain. (2013) 6:52. doi: 10.1186/1756-6606-6-52, 24305089 PMC3907076

[49] Endo T. Glycobiology of α-dystroglycan and muscular dystrophy. J Biochem. (2015) 157:1–12. doi: 10.1093/jb/mvu066 25381372

[50] Patricia LH Christina L Lynne W Andrew Edmondson A. Biochemical testing for congenital disorders of glycosylation: a technical standard of the American College of Medical Genetics and Genomics (ACMG). Genet Med. (2025) 27:101328. doi: 10.1016/j.gim.2024.101328 39945761

[51] Altassan R Péanne R Jaeken J Barone R Bidet M Borgel D . International clinical guidelines for the management of phosphomannomutase 2-congenital disorders of glycosylation: diagnosis, treatment and follow up. J Inherit Metab Dis. (2019) 42:5–28. doi: 10.1002/jimd.12024, 30740725

[52] Shah R Eklund EA Radenkovic S Sadek M Shammas I Verberkmoes S . ALG13-congenital disorder of glycosylation (ALG13-CDG): updated clinical and molecular review and clinical management guidelines. Mol Genet Metab. (2024) 142:108472. doi: 10.1016/j.ymgme.2024.108472, 38703411 PMC11402470

[53] Ng BG Eklund EA Shiryaev SA Dong YY Abbott MA Asteggiano C . Predominant and novel de novo variants in 29 individuals with ALG13 deficiency: clinical description, biomarker status, biochemical analysis, and treatment suggestions. J Inherit Metab Dis. (2020) 43:1333–48. doi: 10.1002/jimd.12290, 32681751 PMC7722193

[54] Monticelli M D'Onofrio T Jaeken J Morava E Andreotti G Cubellis MV. Congenital disorders of glycosylation: narration of a story through its patents. Orphanet J Rare Dis. (2023) 18:247. doi: 10.1186/s13023-023-02852-w 37644541 PMC10466741

[55] Boyer SW Johnsen C Morava E. Nutrition interventions in congenital disorders of glycosylation. Trends Mol Med. (2022) 28:463–81. doi: 10.1016/j.molmed.2022.04.003, 35562242 PMC9375550

[56] Lindsey BG Manisha P Jong MR. Ketogenic diets, mitochondria, and neurological diseases. J Lipid Res. (2014) 55:2211–28. doi: 10.1194/jlr.R048975 24847102 PMC4617125

[57] Akella NM Ciraku L Reginato MJ. Fueling the fire: emerging role of the hexosamine biosynthetic pathway in cancer. BMC Biol. (2019) 17:52. doi: 10.1186/s12915-019-0671-3, 31272438 PMC6610925

[58] Shimazu T Hirschey MD Newman J He W Shirakawa K Le Moan N . Suppression of oxidative stress by β-hydroxybutyrate, an endogenous histone deacetylase inhibitor. Science. (2013) 339:211–4. doi: 10.1126/science.1227166 23223453 PMC3735349

[59] Arslan N Guzel O Kose E Yılmaz U Kuyum P Aksoy B . Is ketogenic diet treatment hepatotoxic for children with intractable epilepsy? Seizure. (2016) 43:32–8. doi: 10.1016/j.seizure.2016.10.024, 27866088

[60] Dressler A Chiara H Benninger F Waldhoer T Gröppel G Trimmel-Schwahofer P . Effects of the ketogenic diet on platelet counts and global coagulation tests in childhood epilepsy. Seizure. (2020) 80:31–7. doi: 10.1016/j.seizure.2020.03.017, 32512283

[61] van der Louw E van den Hurk D Neal E Leiendecker B Fitzsimmon G Dority L . Ketogenic diet guidelines for infants with refractory epilepsy. Eur J Paediatr Neurol. (2016) 20:798–809. doi: 10.1016/j.ejpn.2016.07.009, 27470655

[62] Kossoff EH Zupec-Kania BA Auvin S Ballaban-Gil KR Christina Bergqvist AG Blackford R . Optimal clinical management of children receiving dietary therapies for epilepsy: updated recommendations of the international ketogenic diet study group. Epilepsia Open. (2018) 3:175–92. doi: 10.1002/epi4.12225 29881797 PMC5983110

[63] Zhong M Balakrishnan B Guo AJ Lai K. AAV9-based PMM2 gene replacement augments PMM2 expression and improves glycosylation in primary fibroblasts of patients with phosphomannomutase 2 deficiency (PMM2-CDG). Mol Genet Metab Rep. (2024) 38:101035. doi: 10.1016/j.ymgmr.2023.101035, 38130891 PMC10733668

[64] Pérez B Rodríguez-Pascau L Vilageliu L Grinberg D Ugarte M Desviat LR. Present and future of antisense therapy for splicing modulation in inherited metabolic disease. J Inherit Metab Dis. (2010) 33:397–403. doi: 10.1007/s10545-010-9135-1, 20577904

[65] Yuste-Checa P Medrano C Gámez A Desviat LR Matthijs G Ugarte M . Antisense-mediated therapeutic pseudoexon skipping in TMEM165-CDG. Clin Genet. (2015) 87:42–8. doi: 10.1111/cge.12402, 24720419

[66] Datta AN Bahi-Buisson N Bienvenu T Buerki SE Gardiner F Cross JH . The phenotypic spectrum of X-linked, infantile onset ALG13-related developmental and epileptic encephalopathy. Epilepsia. (2021) 62:325–34. doi: 10.1111/epi.16761, 33410528 PMC7898319

[67] Ng BG Underhill HR Palm L Bengtson P Rozet JM Gerber S . DPAGT1 deficiency with encephalopathy (DPAGT1-CDG): clinical and genetic description of 11 new patients. JIMD Rep. (2019) 44:85–92. doi: 10.1007/8904_2018_128 30117111 PMC6323016

[68] Park JH Marquardt T. Treatment options in congenital disorders of glycosylation. Front Genet. (2021) 12:735348. doi: 10.3389/fgene.2021.735348, 34567084 PMC8461064

[69] Cruz V Xin B Wang H. GM3 synthase deficiency. In: Adam MP Bick S Mirzaa GM Pagon RA Wallace SE Amemiya A, editors. GeneReviews(®). Seattle: University of Washington (2023). Available online at: https://ncbi.nlm.nih.gov/books/NBK593237/37471511

[70] Muffels IJJ Sadek M Kozicz T Morava E. Assessing age of onset and clinical symptoms over time in patients with heterozygous pathogenic DHDDS variants. J Inherit Metab Dis. (2024) 47:935–44. doi: 10.1002/jimd.12769 39540616

[71] Aeby A Prigogine C Vilain C Malfilatre G Jaeken J Lederer D . RFT1-congenital disorder of glycosylation (CDG) syndrome: a cause of early-onset severe epilepsy. Epileptic Disord. (2016) 18:92–6. doi: 10.1684/epd.2016.0802, 26892341

[72] Barba C Darra F Cusmai R Procopio E Dionisi Vici C Keldermans L . Congenital disorders of glycosylation presenting as epileptic encephalopathy with migrating partial seizures in infancy. Dev Med Child Neurol. (2016) 58:1085–91. doi: 10.1111/dmcn.13141, 27172925

[73] Levy RJ Frater CH Gallentine WB Phillips JM Ruzhnikov MR. Delineating the epilepsy phenotype of NGLY1 deficiency. J Inherit Metab Dis. (2022) 45:571–83. doi: 10.1002/jimd.12494, 35243670

[74] Bellai-Dussault K Nguyen TTM Baratang NV Jimenez-Cruz DA Campeau PM. Clinical variability in inherited glycosylphosphatidylinositol deficiency disorders. Clin Genet. (2019) 95:112–21. doi: 10.1111/cge.13425, 30054924

[75] Bögershausen N Cavdarli B Nagai TH Milev MP Wolff A Mehranfar M . SEC24C deficiency causes trafficking and glycosylation abnormalities in an epileptic encephalopathy with cataracts and dyserythropoeisis. JCI Insight. (2025) 10:e173484. doi: 10.1172/jci.insight.173484, 40131364 PMC12128993

[76] Rymen D Lindhout M Spanou M Ashrafzadeh F Benkel I Betzler C . Expanding the clinical and genetic spectrum of CAD deficiency: an epileptic encephalopathy treatable with uridine supplementation. Genet Med. (2020) 22:1589–97. doi: 10.1038/s41436-020-0933-z, 32820246

[77] Shwetabh RK Singh A Mandal K Naranje K Verma A. Congenital disorder of glycosylation type IIb in an infant with developmental and epileptic encephalopathy. BMJ Case Rep. (2025) 18:e268902. doi: 10.1136/bcr-2025-268902 PMC12587477 41192964

[78] Takuya H Yoshinao W Tomoko M Machiko K Yohei M Yumiko O . Novel ALG12 variants and hydronephrosis in siblings with impaired N-glycosylation. Brain Dev. (2021) 43:945–51. doi: 10.1016/j.braindev.2021.05.013 34092405

[79] Alaamery M Massadeh S Aldarwish M Albesher N Aljawini N Alahmed O . Case report: a founder UGDH variant associated with developmental epileptic encephalopathy in Saudi Arabia. Front Genet. (2024) 14:14. doi: 10.3389/fgene.2023.1294214, 38292436 PMC10824937

[80] den Hollander B Rasing A Post MA Klein WM Oud MM Brands MM . NANS-CDG: delineation of the genetic, biochemical, and clinical Spectrum. Front Neurol. (2021) 12:668640. doi: 10.3389/fneur.2021.668640, 34163424 PMC8215539

[81] Zilmer M Edmondson AC Khetarpal SA Alesi V Zaki MS Rostasy K . Novel congenital disorder of O-linked glycosylation caused by GALNT2 loss of function. Brain. (2020) 143:1114–26. doi: 10.1093/brain/awaa063, 32293671 PMC7534148

[82] Shijia O Ting W Xiaojuan T Zeyong D Quanzhen T Ying Y . Genotype and phenotype spectrum of epilepsy patients with congenital disorders of glycosylation associated with GPAA1 variants. Seizure. (2026) 138:93–102. doi: 10.1016/j.seizure.2026.03.018 42001530

[83] Dardas Z Harrold L Calame DG Salter CG Kikuma T Guay KP . Bi-allelic UGGT1 variants cause a congenital disorder of glycosylation. Am J Hum Genet. (2025) 112:1139–57. doi: 10.1016/j.ajhg.2025.03.018, 40267907 PMC12120171

[84] Silvia R Diego M Yuebo Z Graeme JP Arianna M Alessandra T . TRAPPC9-CDG: a novel congenital disorder of glycosylation with dysmorphic features and intellectual disability. Genet Med. (2022) 24:894–904. doi: 10.1016/j.gim.2021.12.012 35042660 PMC13734748

[85] Liu Z Wang Y Yang F Yang Q Mo X Burstein E . GMPPB-congenital disorders of glycosylation associate with decreased enzymatic activity of GMPPB. Mol Biomed. (2021) 2:13. doi: 10.1186/s43556-021-00027-2, 35006422 PMC8607393

[86] Li R Yang J Ma J Zhang A Li H. Case report: novel NUS1 variant in a Chinese patient with tremors and intellectual disability. Front Genet. (2024) 15:1373448. doi: 10.3389/fgene.2024.1373448 38655050 PMC11035736

[87] Dulce Q Jaak J. Treatment of congenital disorders of glycosylation: an overview. Mol Genet Metab. (2024) 143:108567. doi: 10.1016/j.ymgme.2024.108567 39236565

[88] Swaroop S Dasgupta S Srivastava P Jain SD Nag DS Tanti SK. Neuro-ophthalmic presentation of steroid 5a-reductase type 3 congenital disorder of glycosylation: a case of monozygotic twins. Cureus. (2026) 18:e102557. doi: 10.7759/cureus.102557, 41769439 PMC12949591

[89] Al Tuwaijri A Alyafee Y Umair M Alsubait A Alharbi M AlEidi H . Congenital disorder of glycosylation with defective fucosylation 2 (FCSK gene defect): the third report in the literature with a mild phenotype. Mol Genet Genomic Med. (2023) 11:e2117. doi: 10.1002/mgg3.2117, 36426412 PMC10094070

[90] Ng BG Zhang W Neil JE Danish M Marafi D Kamal TM . A homozygous nonsense variant in the oligosaccharyltransferase complex gene, RPN1, causes a congenital disorder of glycosylation. HGG Adv. (2026) 7:100604. doi: 10.1016/j.xhgg.2026.100604, 41935956 PMC13112475

[91] Witters P. Tahata S. Barone R. Ounap K. Salvarinova R. Gronborg S. Clinical and biochemical improvement with galactose supplementation in SLC35A2-CDG. Genet Med (2020) 22:1102–1107. doi: 10.1038/s41436-020-0767-8 32103184 PMC7275909

[92] Cirnigliaro L Bianchi P Sturiale L Garozzo D Mangili G Keldermans L . COG6-CDG: novel variants and novel malformation. Birth Defects Res. (2022) 114:165–74. doi: 10.1002/bdr2.1981, 35068072 PMC9306771

[93] Olivier F Gilbert V David MS Sarah BM. Progressive loss of cerebral structures in ALG11-related congenital disorder of glycosylation. Pediatr Neurol. (2025) 164:7–9. doi: 10.1016/j.pediatrneurol.2024.12.009 39809108

[94] Foulquier F Amyere M Jaeken J Zeevaert R Schollen E Race V . TMEM165 deficiency causes a congenital disorder of glycosylation. Am J Hum Genet. (2012) 91:15–26. doi: 10.1016/j.ajhg.2012.05.002, 22683087 PMC3397274

[95] Qureshi S Raza Alvi J Mehmood A Sultan T. Unraveling COG-4 gene associated epileptic encephalopathy: a rare congenital disorder of glycosylation (CDG-IIj). J Int Child Neurol Assoc. (2026) 1. doi: 10.17724/jicna.2025.298

[96] Zhao L Zeng L Yi M Yuan W. Early neonatal diagnosis of SSR4-related congenital disorder of glycosylation with severe congenital heart defects: a case report and systematic review. Front Pediatr. (2026) 14:1780997. doi: 10.3389/fped.2026.1780997, 41960028 PMC13057274

[97] Francisco R Brasil S Poejo J Jaeken J Pascoal C Videira PA . Congenital disorders of glycosylation (CDG): state of the art in 2022. Orphanet J Rare Dis. (2023) 18:329. doi: 10.1186/s13023-023-02879-z, 37858231 PMC10585812

[98] Verheijen J Tahata S Kozicz T Witters P Morava E. Therapeutic approaches in congenital disorders of glycosylation (CDG) involving N-linked glycosylation: an update. Genet Med. (2020) 22:268–79. doi: 10.1038/s41436-019-0647-2, 31534212 PMC8720509

[99] Čechová A Altassan R Borgel D Bruneel A Correia J Girard M . Consensus guideline for the diagnosis and management of mannose phosphate isomerase-congenital disorder of glycosylation. J Inherit Metab Dis. (2020) 43:671–93. doi: 10.1002/jimd.12241 32266963 PMC7574589

[100] Madani S Sayarifard F Tajdini P Mohsenipour R Khoram Khorshid HR Rezaei N. Novel treatment for congenital disorder of glycosylation in a patient with novel homozygote mutation of PMM2: a case report and review literature. Endocr Metab Immune Disord Drug Targets. (2021) 21:2296–9. doi: 10.2174/1871530321666210415105917, 33858316

[101] Muffels IJJ Kozicz T Perlstein EO Morava E. The therapeutic future for congenital disorders of glycosylation. J Inherit Metab Dis. (2025) 48:e70011. doi: 10.1002/jimd.70011 40064184 PMC13694125

[102] Budhraja R Radenkovic S Jain A Muffels IJJ Ismaili MHA Kozicz T . Liposome-encapsulated mannose-1-phosphate therapy improves global N-glycosylation in different congenital disorders of glycosylation. Mol Genet Metab. (2024) 142:108487. doi: 10.1016/j.ymgme.2024.108487, 38733638 PMC11166087

[103] Almeida Antonio M Murakami Y Baker A Maeda Y Roberts IAG Kinoshita T . Targeted therapy for inherited GPI deficiency. N Engl J Med. (2007) 356:1641–7. doi: 10.1056/NEJMoa063369 17442906

[104] Gámez A Serrano M Gallego D Vilas A Pérez B. New and potential strategies for the treatment of PMM2-CDG. Biochim Biophys Acta Gen Subj. (2020) 1864:129686. doi: 10.1016/j.bbagen.2020.129686, 32712172

[105] Jehan M Larissa V Anab M Diederik Eva M. Acetazolamide treatment in late onset CDG type 1 due to biallelic pathogenic DHDDS variants. Mol Genet Metab Rep. (2022) 32:100901. doi: 10.1016/j.ymgmr.2022.100901 36046393 PMC9421445

